# Microsatellite-stable diploid carcinoma: a biologically distinct and aggressive subset of sporadic colorectal cancer

**DOI:** 10.1054/bjoc.2000.1554

**Published:** 2001-01

**Authors:** N J Hawkins, I Tomlinson, A Meagher, R L Ward

**Affiliations:** School of Pathology, University of NSW, Sydney, Australia; Molecular and Population Genetics, Imperial Cancer Research Fund, Lincoln's Inn Fields, London, WC2A 3PX, UK; School of Medicine, University of NSW, Sydney, Australia; Departments of Medical Oncology, Colorectal Surgery, St Vincent's Hospital, Victoria St, Darlinghurst, 2010, Australia

**Keywords:** colorectal carcinoma, ploidy, survival, microsatellite instability

## Abstract

Chromosomal instability and microsatellite instability represent the major pathways for colorectal cancer (CRC) progression. However, a significant percentage of CRC shows neither pattern of instability, and thus represents a potentially distinctive form of the disease. Flow cytometry was used to determine the degree of DNA aneuploidy in 46 consecutive sporadic colorectal cancers. Microsatellite status was determined by PCR amplification using standard markers, while immunostaining was used to examine the expression of *p53*. K- *ras* status was determined by restriction-mediated PCR assay. Twenty-five (54%) tumours were aneuploid, 14 (30%) were diploid and microsatellite-stable and seven (15%) were diploid and microsatellite-unstable. Tumours with microsatellite instability were more likely to be right sided, to occur in women and to be associated with an improved survival. Aneuploid tumours were significantly more common in men and were likely to be left sided. The diploid microsatellite-stable (MSS) tumours did not show a sex or site predilection, but were strongly associated with the presence of metastatic disease at the time of diagnosis. Our data suggests that diploid, MSS tumours represent a biologically and phenotypically distinct subset of colorectal carcinoma, and one that is associated with the early development of metastases. We suggest that the genetic stability that characterizes these tumours may favour the maintenance of an invasive phenotype, and thus facilitate disease progression. These findings may have important implications for treatment options in this disease subset. © 2001 Cancer Research Campaign http://www.bjcancer.com


*British Journal of Cancer* (2001) **84**(2), 232–236
© 2001 Cancer Research Campaign
doi: 10.1054/ bjoc.2000.1554, available online at http://www.idealibrary.com on IDEAL®

http://www.bjcancer.com

### Microsatellite-stable diploid carcinoma: a biologically distinct and aggressive subset of sporadic colorectal cancer

**NJ Hawkins[1], I Tomlinson[2], A Meagher[3] and RL Ward[4,5]**

[1]School of Pathology, University of NSW, Sydney, Australia; [2]Molecular and Population Genetics, Imperial Cancer Research Fund, Lincoln's Inn Fields, London WC2A 3PX, UK; [3]School of Medicine, University of NSW, Sydney, Australia; [4]Departments of Medical Oncology and [5]Colorectal Surgery, St Vincent's Hospital, Victoria St, Darlinghurst 2010, Australia

**Summary** Chromosomal instability and microsatellite instability represent the major pathways for colorectal cancer (CRC) progression. However, a significant percentage of CRC shows neither pattern of instability, and thus represents a potentially distinctive form of the disease. Flow cytometry was used to determine the degree of DNA aneuploidy in 46 consecutive sporadic colorectal cancers. Microsatellite status was determined by PCR amplification using standard markers, while immunostaining was used to examine the expression of *p53*. K-*ras* status was determined by restriction-mediated PCR assay. Twenty-five (54%) tumours were aneuploid, 14 (30%) were diploid and microsatellite-stable and seven (15%) were diploid and microsatellite-unstable. Tumours with microsatellite instability were more likely to be right sided, to occur in women and to be associated with an improved survival. Aneuploid tumours were significantly more common in men and were likely to be left sided. The diploid microsatellite-stable (MSS) tumours did not show a sex or site predilection, but were strongly associated with the presence of metastatic disease at the time of diagnosis. Our data suggests that diploid, MSS tumours represent a biologically and phenotypically distinct subset of colorectal carcinoma, and one that is associated with the early development of metastases. We suggest that the genetic stability that characterizes these tumours may favour the maintenance of an invasive phenotype, and thus facilitate disease progression. These findings may have important implications for treatment options in this disease subset. © 2001 Cancer Research Campaign http://www.bjcancer.com

**Keywords**: colorectal carcinoma; ploidy; survival; microsatellite instability

Over the last decade, there has been a great increase in our understanding of the genetic basis of colorectal tumour development (Martin et al, 1999). In particular, there has been recognition of a distinct pathway of colorectal carcinogenesis, characterized by sporadic or hereditary defects in DNA repair enzymes, and defined genetically by microsatellite instability (Ionov et al, 1993; Peltomaki et al, 1993; Thibodeau et al, 1993; 1998; Herman et al, 1998; Velgl et al, 1998). This new knowledge has thrown into relief the more typical pathway of colorectal cancer (CRC) progression, characterized by mutations of *p53* and chromosomal aneuploidy, a phenomenon often referred to as chromosomal instability (Cianchi et al, 1999). Aneuploidy is detected in 60–90% of colorectal cancers, and in many (Witzig et al, 1991; Tsuchiya et al, 1992; Lanza et al, 1998; Pietra et al, 1998; Flyger et al, 1999) but not all studies (Visscher et al, 1990; Cohn et al, 1997; Tonouchi et al, 1998) has been associated with worse disease outcome.

It has been widely assumed that either chromosomal or microsatellite instability pathways underly colorectal tumour development. However, a recent study has demonstrated that a percentage of colorectal carcinomas show neither pattern of genetic instability, and may represent a potentially distinctive form of the disease (Georgiades et al, 1999). The authors postulated that these seemingly genetically stable tumours may have developed through an as yet uncharacterized unique molecular pathway.

*Received 6 July 2000*
*Accepted 4 October 2000*

*Correspondence to:* R Ward. E-mail: r.ward@garvan.unsw.edu.au

In this report, we have classified apparently sporadic colorectal carcinomas according to the presence or absence of both chromosomal and microsatellite instability, and have assessed the clinicopathological features of those tumours characterized by both chromosomal and microsatellite stability.

### MATERIALS AND METHODS

### Patient recruitment and follow-up

After obtaining informed consent, 43 consecutive individuals undergoing surgical resection of adenocarcinoma of the colon or rectum at St Vincent's Hospital, Sydney, were enrolled in this study. Individuals were excluded where preoperative radio or chemotherapy had been administered. Patients with inflammatory bowel disease, or with a known history of familial adenomatous polyposis (FAP) or hereditary non-polyposis coli (HNPCC) were also excluded from this study, as were those individuals where the primary tumour was incompletely resected (R1 or R2) (Hermanek and Sobin, 1998). Fresh representative tissue samples (500 µg) from all tumours and paired normal colonic mucosa were used immediately for flow cytometric analysis, or stored at –70˚C for DNA extraction.

### Histopathological analysis of tumours

For all tumours, the histopathological type, stage and size of the tumour were determined independently by a histopathologist within the Department of Anatomical Pathology, St Vincent's Hospital.

The tumour grade, extent of mucin production, tumour growth pattern, and presence of intraepithelial lymphocytes, were determined prospectively without knowledge of the mismatch repair status. Tumours in which less than 10% of cells formed glands were classified as high-grade (poorly differentiated), while those containing more than 50% extracellular mucin were classified as mucinous (Jass and Sobin, 1989). The tumour growth pattern was interpreted as either infiltrative or expansile, as per previously published criteria (Jass et al, 1996). Intraepithelial lymphocytes were identified by light microscopy on haematoxylin and eosin sections as cells with the morphology of lymphocytes, seen wholly within tumour epithelium. They were classified as conspicuous when more than 30 were present per 10 high-power fields.

### DNA flow cytometry

Tumour tissue was minced in cold PBS, filtered through a nylon mesh (160 µm) and then centrifuged at 400 ***g*** for 10 min. The cell concentration was adjusted to 5–10 × $10^6$ $ml^{-1}$ and 0.5 ml was added to an equal volume of propidium iodine (PI) detergent solution (100 µg $ml^{-1}$ PI, 1% Triton X 100, 1 µg $ml^{-1}$ RNAse) for 30 min at 4˚C. The suspension was again filtered and the DNA content of the nuclei was measured by means of an Epics XL-MCL (Coulter Electronics, FL, USA) using Coulter system II then multiplus software (Phoenix Technologies, San Diego, USA) for data acquisition and analysis. All tumour samples were run in duplicate, together with nuclei from normal colonic mucosa of the same patient, and with cryopreserved peripheral blood mononuclear cells. Adenocarcinomas with a single G0/G1 peak were classified as DNA diploid, while tumours with at least two separate peaks were considered aneuploid (Hiddemann et al, 1984). The degree of DNA aneuploidy was expressed as the DNA index (DI), which represents the ratio of the modal channel position of the G0/G1 peak of the aneuploid cell population to the diploid reference cells. Tumours with DI between 0.09 and 1.1 were considered near-diploid. In all analysis, the coefficient of variation (CV) of the diploid G0/G1 peaks of tumours as well as normal samples was less than 5%.

### Microsatellite analysis

For preparation of DNA, the frozen tissue was macerated in 500 µl of ice-cold lysis buffer (10 mM Tris-HCl, 1 mM ethylenediamine tetra-acetic acid (EDTA), 100 mM NaCl, 1% sodium dodecyl sulphate (SDS), 500 µg $ml^{-1}$ proteinase K), using a sterile Eppendorf homogenizer. Following incubation overnight with shaking at 50˚C, DNA was extracted with phenol/chloroform, and precipitated with ethanol.

In all reactions, DNA was amplified in a 10 µl volume containing 100 ng of DNA, 200 µM dNTPs, 1.5 mM $MgCl_2$, 0.27 µM of each primer, 0.25 U Tth polymerase in a buffer of 16.6 mM $(NH4)_2SO_4$, 0.45% Triton X 100, 0.2 mg $ml^{-1}$ gelatine, 67 mM Tris-HCl, pH 8.8 (Biotech International Ltd, Western Australia). The reactions were incubated at 95˚C for 5 min, followed by 35 cycles of 95˚C, 57˚C and 72˚C for 1 min each. The primers used in this study were Bat25, Bat26, D5S346, D2S123 and DI7S250 (Boland et al, 1998). Products were run on an ABI 377 sequencer (Foster City, California, USA) and analysed using Genescan and Genotyper software.

Microsatellite instability (MSI) was defined as either a marked alteration in repeat length or as a new discrete band above or below the expected allele. A single observer (RW) performed the analysis of MSI, and equivocal samples were then re-evaluated by a second observer (IT). The results were reported without knowledge of pathological or immunohistochemical status. A tumour sample was considered to show microsatellite instability (MSI) if two or more of the markers demonstrated instability. In all other cases, the tumour was classified as microsatellite stable (MSS).

### Analysis of somatic changes in *p53* and K-*ras* genes

Mutations at the first and second bases of codon 12 of the K-*ras* gene were detected using REMS-PCR as previously described (Ward et al, 1998). For the identification of accumulation of *p53* within tumour cells, paraffin sections of tumour tissue were subjected to immunohistochemical analysis of *p53* as previously described (Ward et al, 1997), using the mouse anti-human *p53* antibody DO7 (DAKO, High Wycombe, UK). Tumour was considered to show accumulation of *p53* protein when more than 20% of tumour cells showed nuclear staining of moderate to high intensity, in the absence of staining in the stromal cells and normal epithelium.

### Statistical analysis

Survival was measured from the date of resection of colorectal cancer until death or until the cansor date of 1 March 2000. Time to recurrence was the period from resection to medical documentation of tumour recurrence. Survival and time to recurrence were censored at time of death for patients dying of causes other than colorectal cancer. Survival curves were prepared according to the method of Kaplan and Meier, and univariate survival distributions were compared by the log-rank test. Categorical variables were compared using the Chi-square test or the Fisher's exact test as appropriate. A probability value of less than 0.05 was considered significant. All data was analysed using the SPSS statistical software V9.0 (SPSS Inc, Chicago, IL, USA).

### RESULTS

A total of 46 fresh tumour specimens were assayed from 27 males and 16 females (ages 54–94, mean 70 ± 9.5 years). Eight of these

**Table 1** Clinical characteristics of diploid microsatellite stable, aneuploid and diploid microsatellite-unstable tumours. Probability was calculated using Pearson chi-squared

| | Diploid MSS | Diploid MSI | Aneuploid | Probability |
|---|---|---|---|---|
| Age | | | | |
| Mean | 68.6 | 78.2 | 69.3 | *P* = 0.07 |
| 95% CI | 63.5–73.8 | 67.7–88.7 | 65.8–72.9 | |
| Sex | | | | |
| Female | 5 | 6* | 6 | *P* = 0.01 |
| Male | 9 | 1* | 19* | |
| Tumour stage | | | | |
| I | 3 | 2 | 3 | *P* = 0.09 |
| II | 3 | 4 | 9 | |
| III | 3 | 1 | 11 | |
| IV | 5 | 0 | 2 | |
| Total (%) | 14 (30) | 7 (15) | 25 (54) | |

*Factors contributing to a significant correlation, MSS = microsatellite stable, MSI = microsatellite instability, I–IV represent AJCC/UICC tumour stages

© 2001 Cancer Research Campaign 

tumours were TNM stage I, while 16 were stage II, 15 were stage III and seven were stage IV. 21 tumours (45%) were diploid, and seven of these tumours also demonstrated microsatellite instability (Table 1). Since all the aneuploid cancers were microsatellite-stable, the tumours were classified into the following groups; aneuploid (25 of 46, 54%), diploid MSS (14 of 46, 30%) and diploid MSI (7 of 46, 15%) (Table 1). No difference was found in the S-phase fraction among the different groups (diploid MSS = 20.7 ± 24.3, aneuploid = 19.9 ± 10.1, diploid MSI = 10.5 ± 8.8).

There were a number of significant differences in the clinicopathological characteristics of the three groups of tumours (Tables 1 and 2). The diploid MSI tumours were significantly more likely to occur in women, while aneuploid tumours were more likely to arise in men. Diploid MSI tumours were more likely to be right-sided tumours, and to harbour increased numbers of intraepithelial lymphocytes, while there were more left-sided lesions in the aneuploid group. However, the diploid MSS group showed no association with sex, tumour sidedness, or the extent of lymphocytic infiltration within the tumour.

K-*ras* mutations at codon 12 were detected in 13 of the 44 tumours analysed (30%), and were less frequent in diploid MSI tumours (0%) than in stable tumours (54%). Immunohistochemical evidence of p53 accumulation was observed in 17 of 46 tumours (37%), and was much less common in diploid MSI cancers (0%) than in either diploid MSS (55%) or aneuploid tumours (92%). In both cases, these differences did not reach statistical significance (Table 2).

The presence of metastatic disease at the time of diagnosis was significantly more common in individuals with diploid MSS tumours (5 of 14, 35%) than in those with aneuploid (2 of 25, 8%) or diploid MSI tumours (0 of 7) ($P$ = 0.03). However, there was no significant correlation between the T or N stage of the tumour and ploidy/microsatellite status. Comparison of the TNM stage of the diploid MSS group with the other tumours (aneuploid and diploid MSI groups combined) further demonstrated the association of the former with Stage IV disease at presentation ($P$ = 0.01).

The overall survival curves for patients with all-stage cancers according to ploidy/microsatellite status are shown in Figure 1. Despite the fact that individuals with diploid MSS tumours were more likely to have metastatic disease at the time of diagnosis, there was no difference in outcome between this group and individuals with aneuploid tumours, either in terms of overall survival or time to recurrence. In contrast, the diploid MSI group showed a trend towards improved overall survival in comparison to the other groups, and in fact, only one of the seven individuals with a diploid MSI tumour developed recurrent disease. However, this difference did not reach statistical significance. The sites of recurrence did not show any differences between the three ploidy/microsatellite groups.

**Table 2** Pathological and genetic characteristics of sporadic colorectal cancers, based on ploidy and microsatellite analysis

| Tumour characteristics | Diploid MSS | Aneuploid | Diploid MSI | Probability |
|---|---|---|---|---|
| Tumour site | | | | |
| Right | 5 | 5* | 6* | $P$ = 0.01 |
| Left | 9 | 20* | 1* | |
| Intraepithelial ymphocytes | | | | |
| I Increased | 2 | 3 | 4* | $P$ = 0.02 |
| Normal | 12 | 22 | 3* | |
| Tumour differentiation | | | | |
| Poor | 1 | 2 | 0 | $P$ = 0.75 |
| Moderate | 13 | 23 | 7 | |
| Mucinous phenotype | | | | |
| Yes | 3 | 5 | 2 | $P$ = 0.88 |
| No | 11 | 20 | 5 | |
| Tumour margins | | | | |
| Circumscribed | 8 | 18 | 7 | $P$ = 0.12 |
| Infiltrative | 6 | 7 | 0 | |
| K-*ras* status (codon 12) | | | | |
| Mutant | 3 | 10 | 0 | $P$ = 0.16 |
| Wild-type | 11 | 13 | 7 | |
| p53 protein amount (IHC) | | | | |
| Increased | 5 | 12 | 0 | $P$ = 0.06 |
| Normal | 9 | 13 | 7 | |

Probability was calculated using Pearson chi-squared. [a]Factors contributing to a significant correlation, MSS = microsatellite-stable, MSI = microsatellite instability, IHC = immunohistochemistry

### DISCUSSION

Over many years, a large number of studies have examined the relationship between nuclear DNA content and clinicopathological variables in colorectal carcinoma. A number of these studies have suggested that those tumours with diploid DNA content have a distinctive morphological appearance and a less aggressive clinical behaviour (Ruschoff et al, 1997; Forster et al, 1998; Lanza et al, 1998, 1999), yet many other studies have presented contrary results (Cohn et al, 1997; Tonouchi et al, 1998). Such discrepancies have largely been attributed to methodological variations related to the type of tissue used and the manner of its collection (Bauer et al, 1993).

In this study we have demonstrated that colorectal cancer can be classified into three distinct groups on the basis of the microsatellite and DNA ploidy status (aneuploid, diploid MSS and diploid MSI). Many of the observations made in this study regarding the frequency, phenotype and prognosis of microsatellite-unstable tumours are in concordance with previously published studies (Kim et al, 1994; Jass et al, 1998; Halling et al, 1999). These MSI tumours are more frequent in the right colon and intraepithelial

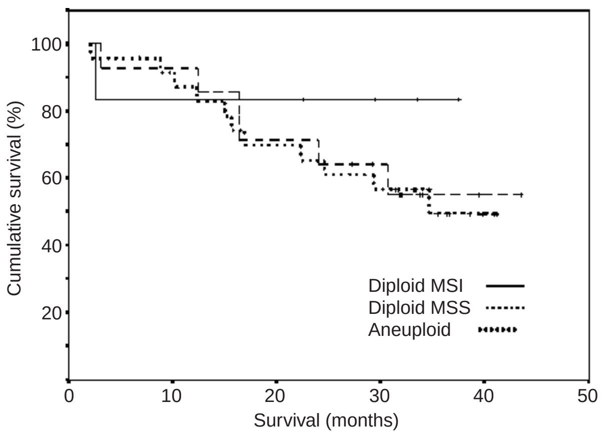

**Figure 1** Overall survival plotted according to ploidy/microsatellite status. There was no significant difference between the groups ($P$ = 0.53)

*© 2001 Cancer Research Campaign*

and peritumoural lymphocytic infiltration was often prominent. Like others, we have found that MSI tumours are more common in women, that there is a strong correlation between MSI and the absence of *p53* mutations as determined by immunostaining, and that individuals with such tumours may have an improved clinical outcome (Fujiwara et al, 1998; Elsaleh et al, 2000).

Clearly, many of the diploid tumours examined in previous studies contain a high percentage of these diploid MSI tumours. Yet they also contain a distinct subgroup of tumours that are both diploid and microsatellite-stable. These diploid MSS tumours share few of the distinctive clinicopathological features of the diploid MSI group, and on this basis clearly represent a distinct class of colorectal carcinoma. It is apparent that diploid MSS tumours are equally likely to occur in males as females and that they originate throughout the colon.

However, the most striking difference between diploid MSS and diploid MSI tumours relates to their pattern of invasion and clinical behaviour. While all diploid MSI tumours demonstrate a circumscribed growth pattern, nearly half of the diploid MSS tumours display an infiltrative growth pattern. Likewise, over one third of individuals with diploid MSS tumours have metastases present at the time of diagnosis whereas distant spread is not seen in any of the diploid MSI tumours, and in only 8% of the aneuploid tumours. These observations, together with the survival data, provide strong support for the argument that diploid MSS tumours represent an aggressive subset of colorectal carcinomas. Furthermore, this data suggests a plausible explanation for the longstanding confusion in the literature regarding the prognostic significance of aneuploidy, for in all studies to date, the grouping of diploid tumours includes two biologically distinct tumour types, with markedly different outcomes.

It seems paradoxical that tumours with neither chromosomal or microsatellite instability display apparently early progression, with an infiltrative phenotype and widespread dissemination at the time of presentation. One possible explanation may come from the fact that, by definition, all carcinomas develop an invasive phenotype in their progression from adenomas. It is possible that the unique genetic stability of MSS diploid tumours makes them better able to maintain an invasive phenotype than either aneuploid or diploid MSI tumours.

As well as allowing maintenance of a particular phenotype, the unique genetic stability of diploid MSS tumours could also be predicted to make those tumours more susceptible to changes in their environment. In this regard, it is interesting to note that there are very few established colorectal carcinoma cell lines which are both diploid and microsatellite-stable (Eshleman et al, 1998). Furthermore, Wylie and colleagues recently noted that it was not possible to establish tumour xenografts in nude mice using MSI-stable, low chromosomal instability tumours (Georgiades et al, 1999). These observations support the hypothesis that genetic stability lessens the ability of these tumour cells to develop the biochemical machinery requisite for growth in an altered environment, either in vitro or in vivo.

This hypothesis may have considerable importance in terms of research based on cell culture models of human cancer. For not only will their be a total absence of diploid MSS cells in any of these studies, but it is possible that these cells would represent a far more relevant source for the examination of the biology of invasion, at least as it pertains to colorectal cancer.

Of more direct clinical relevance, an inability to respond to adverse environmental stressors has clear implications for the success of chemotherapy in these tumours. It has recently been shown that MSI tumours show a good response to chemotherapy (Elsaleh et al, 2000), yet in theory it is possible that diploid MSS tumour cells may be more susceptible to chemotherapeutic agents, since resistant clones would be less likely to emerge. We can provide only limited data on the behaviour of these tumours, by noting that palliative chemotherapy was administered to all five individuals who presented with stage IV diploid MSS disease and three showed partial responses to treatment. While this is a much higher rate of response than would be expected of colorectal carcinoma in general, the validation of these observations would require a more extensive study.

The results of this study support and extend the observations of Georgiades and colleagues regarding the presence of a distinct subset of colorectal carcinoma with a uniquely stable genotype, and highlight the need for further analysis of this group, particularly in terms of their biological behaviour and possible altered responsiveness to chemotherapy.

### ACKNOWLEDGEMENTS

The authors wish to acknowledge David Wanigesekera from St Vincent's Hospital, Andy Davies and the staff of the Equipment Park, Imperial Cancer Research Fund for their technical assistance and Virgina Nink from the Centre for Immunology, St Vincent's Hospital for flow cytometry analysis. RLW is supported in part by the St Vincent's Clinic Foundation Travelling Fellowship, and by the Macquarie Bank–Royal Australian College of Physicians Fellowship.

### REFERENCES

Bauer KD, Bagwell CB, Giaretti W, Melamed M, Zarbo RJ, Witzig TE and Rabinovitch PS (1993) Consensus review of the clinical utility of DNA flow cytometry in colorectal cancer. *Cytometry* **14**: 486–491

Boland CR, Thibodeau SN, Hamilton SR, Sidransky D, Eshleman JR, Burt RW, Meltzer SJ, Rodriguez-Bigas MA, Fodde R, Ranzani GN and Srivastava S (1998) A National Cancer Institute Workshop on Microsatellite Instability for cancer detection and familial predisposition: development of international criteria for the determination of microsatellite instability in colorectal cancer. *Cancer Res* **58**: 5248–5257

Cianchi F, Balzi M, Becciolini A, Giache V, Messerini L, Palomba A, Tisti E, Faraoni P, Chellini F, Pucciani F, Perigli G and Cortesini C (1999) Correlation between DNA content and p53 deletion in colorectal cancer. *Eur J Surg* **165**: 363–368

Cohn KH, Ornstein DL, Wang F, LaPaix FD, Phipps K, Edelsberg C, Zuna R, Mott LA and Dunn JL (1997) The significance of allelic deletions and aneuploidy in colorectal carcinoma. Results of a 5-year follow-up study. *Cancer* **79**: 233–244

Elsaleh H, Joseph D, Grieu F, Zeps N, Spry N and Iacopetta B (2000) Association of tumour site and sex with survival benefit from adjuvant chemotherapy in colorectal cancer. *Lancet* **355**: 1745–1750

Eshleman JR, Casey G, Kochera ME, Sedwick WD, Swinler SE, Veigl ML, Willson JK, Schwartz S and Markowitz SD (1998) Chromosome number and structure both are markedly stable in RER colorectal cancers and are not destabilized by mutation of p53. *Oncogene* **17**: 719–725

Flyger HL, Larsen JK, Nielsen HJ and Christensen IJ (1999) DNA ploidy in colorectal cancer, heterogeneity within and between tumors and relation to survival. *Cytometry* **38**: 293–300

Forster S, Sattler HP, Hack M, Romanakis K, Rohde V, Seitz G and Wullich B (1998) Microsatellite instability in sporadic carcinomas of the proximal colon: association with diploid DNA content, negative protein expression of p53, and distinct histomorphologic features. *Surgery* **123**: 13–18

Fujiwara T, Stolker JM, Watanabe T, Rashid A, Longo P, Eshleman JR, Booker S, Lynch HT, Jass JR, Green JS, Kim H, Jen J, Vogelstein B and Hamilton SR (1998) Accumulated clonal genetic alterations in familial and sporadic colorectal carcinomas with widespread instability in microsatellite sequences. *Am J Pathol* **153**: 1063–1078

*© 2001 Cancer Research Campaign* 

Georgiades IB, Curtis LJ, Morris RM, Bird CC and Wyllie AH (1999) Heterogeneity studies identify a subset of sporadic colorectal cancers without evidence for chromosomal or microsatellite instability. *Oncogene* **18**: 7933–7940
Halling KC, French AJ, McDonnell SK, Burgart LJ, Schaid DJ, Peterson BJ, Moon-Tasson L, Mahoney MR, Sargent DJ, MJ, OC, Witzig TE, Farr GH, Jr, Goldberg RM and Thibodeau SN (1999) Microsatellite instability and 8p allelic imbalance in stage B2 and C colorectal cancers. *J Natl Cancer Inst* **91**: 1295–1303
Herman JG, Umar A, Polyak K, Graff JR, Ahuja N, Issa JP, Markowitz S, Willson JK, Hamilton SR, Kinzler KW, Kane MF, Kolodner RD, Vogelstein B, Kunkel TA and Baylin SB (1998) Incidence and functional consequences of hMLH1 promoter hypermethylation in colorectal carcinoma. *Proc Natl Acad Sci USA* **95**: 6870–6875
Hermanek P and Sobin LH (1998) TNM. American Joint Committee on Cancer, *Manual for Staging of Cancer*, 5th edn. Lippincott-Raven: Philadelphia
Hiddemann W, Schumann J, Andreef M, Barlogie B, Herman CJ, Leif RC, Mayall BH, Murphy RF and Sandberg AA (1984) Convention on nomenclature for DNA cytometry. Committee on Nomenclature, Society for Analytical Cytology. *Cancer Genet Cytogenet* **13**: 181–183
Ionov Y, Peinado MA, Malkhosyan S, Shibata D and Perucho M (1993) Ubiquitous somatic mutations in simple repeated sequences reveal a new mechanism for colonic carcinogenesis. *Nature* **363**: 558–561
Jass JR and Sobin LH (1989) Histological typing of intestinal tumours. *WHO International Classification of Tumours*, 2nd edn. Springer-Verlag: Berlin
Jass JR, Ajioka Y, Allen JP, Chan YF, Cohen RJ, Nixon JM, Radojkovic M, Restall AP, Stables SR and Zwi LJ (1996) Assessment of invasive growth pattern and lymphocytic infiltration in colorectal cancer. *Histopathology* **28**: 543–548
Jass JR, Do KA, Simms LA, Iino H, Wynter C, Pillay SP, Searle J, Radford-Smith G, Young J and Leggett B (1998) Morphology of sporadic colorectal cancer with DNA replication errors. *Gut* **42**: 673–679
Kim H, Jen J, Vogelstein B and Hamilton SR (1994) Clinical and pathological characteristics of sporadic colorectal carcinomas with DNA replication errors in microsatellite sequences. *Am J Pathol* **145**: 148–156
Lanza G, Gafa R, Santini A, Maestri I, Dubini A, Gilli G and Cavazzini L (1998) Prognostic significance of DNA ploidy in patients with stage II and stage III colon carcinoma: a prospective flow cytometric study. *Cancer* **82**: 49–59
Lanza G, Gafa R, Matteuzzi M and Santini A (1999) Medullary-type poorly differentiated adenocarcinoma of the large bowel: a distinct clinicopathologic entity characterized by microsatellite instability and improved survival. *J Clin Oncol* **17**: 2429–2438
Martin L, Assem M and Piard F (1999) Are there several types of colorectal carcinomas? Correlations with genetic data. *Eur J Cancer Prev* **8**: S13–20
Peltomaki P, Aaltonen LA, Sistonen P, Pylkkanen L, Mecklin JP, Jarvinen H, Green JS, Jass JR, Weber JL, Leach FS and et al. (1993) Genetic mapping of a locus predisposing to human colorectal cancer. *Science* **260**: 810–812
Pietra N, Sarli L, Thenasseril BJ, Costi R, Sansebastiano G and Peracchia A (1998) Risk factors of local recurrence of colorectal cancer: a multivariate study. *Hepatogastroenterology* **45**: 1573–1578
Ruschoff J, Dietmaier W, Luttges J, Seitz G, Bocker T, Zirngibl H, Schlegel J, Schackert HK, Jauch KW and Hofstaedter F (1997) Poorly differentiated colonic adenocarcinoma, medullary type: clinical, phenotypic, and molecular characteristics. *Am J Pathol* **150**: 1815–1825
Thibodeau SN, Bren G and Schaid D (1993) Microsatellite instability in cancer of the proximal colon [see comments]. *Science* **260**: 816–819
Thibodeau SN, French AJ, Cunningham JM, Tester D, Burgart LJ, Roche PC, McDonnell SK, Schaid DJ, Vockley CW, Michels VV, Farr GH, Jr. and O'Connell MJ (1998) Microsatellite instability in colorectal cancer: different mutator phenotypes and the principal involvement of hMLH1. *Cancer Res* **58**: 1713–1718
Tonouchi H, Matsumoto K, Kinoshita T, Itoh H and Suzuki H (1998) Prognostic value of DNA ploidy patterns of colorectal adenocarcinoma: univariate and multivariate analysis. *Dig Surg* **15**: 687–692
Tsuchiya A, Ando Y, Ishii Y, Yoshida T and Abe R (1992) Flow cytometric DNA analysis in Japanese colorectal cancer. A multivariate analysis. *Eur J Surg Oncol* **18**: 585–590
Veigl ML, Kasturi L, Olechnowicz J, Ma AH, Lutterbaugh JD, Periyasamy S, Li GM, Drummond J, Modrich PL, Sedwick WD and Markowitz SD (1998) Biallelic inactivation of hMLH1 by epigenetic gene silencing, a novel mechanism causing human MSI cancers. *Proc Natl Acad Sci USA* **95**: 8698–8702
Visscher DW, Zarbo RJ, Ma CK, Sakr WA and Crissman JD (1990) Flow cytometric DNA and clinicopathologic analysis of Dukes' A & B colonic adenocarcinomas: a retrospective study. *Mod Pathol* **3**: 709–712
Ward RL, Todd AV, Santiago FT, OC and Hawkins NJ (1997) Activation of the K-ras oncogene in colorectal neoplasms is associated with decreased apoptosis. *Cancer* **79**: 1106–1113
Ward RL, Hawkins NJ, O'Grady R, Sheehan CT, OC, Impey H, Roberts N, Fuery C and Todd A (1998) Restriction endonuclease-mediated selective polymerase chain reaction: a novel assay for the detection of K-ras mutations in clinical samples. *Am J Pathol* **153**: 373–379
Witzig TE, Loprinzi CL, Gonchoroff NJ, Reiman HM, Cha SS, Wieand HS, Katzmann JA, Paulsen JK and Moertel CG (1991) DNA ploidy and cell kinetic measurements as predictors of recurrence and survival in stages B2 and C colorectal adenocarcinoma. *Cancer* **68**: 879–888

 *© 2001 Cancer Research Campaign*
